# Predictors of osteoporotic fracture in postmenopausal women: a meta-analysis

**DOI:** 10.1186/s13018-023-04051-6

**Published:** 2023-08-05

**Authors:** Guanghua Long, Chong Liu, Tuo Liang, Zide Zhang, Zhaojie Qin, Xinli Zhan

**Affiliations:** Spine and Osteopathy Ward, The First Affiliated Hospital of Guangxi Medical University, No.6 Shuangyong Road, Nanning, 530021 Guangxi China

**Keywords:** Osteoporotic fracture, Predictors, Postmenopausal women, Meta-analysis

## Abstract

Osteoporosis affects more than 200 million women worldwide, with postmenopausal women being particularly susceptible to this condition and its severe sequelae disproportionately, such as osteoporotic fractures. To date, the current focus has been more on symptomatic treatment, rather than preventive measures. To address this, we performed a meta-analysis aiming to identify potential predictors of osteoporotic fractures in postmenopausal women, with the ultimate goal of identifying high-risk patients and exploring potential therapeutic approaches. We searched Embase, MEDLINE and Cochrane with search terms (postmenopausal AND fracture) AND (“risk factor” OR “predictive factor”) in May 2022 for cohort and case–control studies on the predictors of osteoporotic fracture in postmenopausal women. Ten studies with 1,287,021 postmenopausal women were found eligible for analyses, in which the sample size ranged from 311 to 1,272,115. The surveyed date spanned from 1993 to 2021. Our results suggested that age, BMI, senior high school and above, parity ≥ 3, history of hypertension, history of diabetes mellitus, history of alcohol intake, age at menarche ≥ 15, age at menopause < 40, age at menopause > 50, estrogen use and vitamin D supplements were significantly associated with osteoporotic fracture in postmenopausal women. Our findings facilitate the early prediction of osteoporotic fracture in postmenopausal women and may contribute to potential therapeutic approaches. By focusing on preventive strategies and identifying high-risk individuals, we can work toward reducing the burden of osteoporosis-related fractures in this vulnerable population.

**Supplementary Information:**

The online version contains supplementary material available at 10.1186/s13018-023-04051-6.

## Introduction

Epidemiologic studies have revealed that 11% of the global population is more than 60 years old, and proportion is projected to 22% by the year 2050 [1]. A large proportion of elderly individuals suffers from osteoporosis, a condition that poses various health hazards, including increased morbidity, financial burdens for families and lowered health-related quality of life (HRQoL) [2–5]. Osteoporosis is characterized by abnormal bone microarchitecture and low bone mass, leading to an increased risk of fragility fractures [6, 7]. The combined risk of experiencing any type of clinically concerning fracture in a lifetime is around 40%, which is on par with cardiovascular disease risk [8]. As an important public health problem, osteoporosis is associated with mortality, functional disability and high costs of health system due to the several thousand fractures each year [9]. The aging population is also expected to aggravate the disease burden of osteoporotic fracture [10].

Osteoporosis affects more than 200 million women worldwide. Particularly, postmenopausal women are particularly vulnerable to osteoporosis and its severe sequelae disproportionately, such as osteoporotic fractures [11]. It was estimated that the probability of women over 50 years old affected by osteoporotic fracture was almost one-third [12]. According to the previous literature, the estrogen levels were positively correlated with bone mineral density (BMD) and played a protective role in preventing osteoporotic fractures [13]. This correlation could be explained by the direct impact of estrogen on osteoblast lineage cells, osteocytes and osteoclasts which helps maintain equilibrium between bone formation and resorption [14]. Therefore, the use of estrogen for postmenopausal osteoporosis prevention was approved by the US Food and Drug Administration. However, it is worth noting that while estrogen has shown positive effects on BMD, large treatment trials have yet to conclusively demonstrated its ability to reduce fracture incidence in women with existing osteoporosis [15]. The previous studies also suggested that late menarche age was related with the reduced BMD risk and consequent osteoporosis and osteoporotic fractures [16–18]. Moreover, the shorter reproductive span and earlier menopause were considered as risk factors of osteoporotic fractures [19–21].

One of the key strategies for preventing osteoporotic fractures is to accurately identify the individuals with high osteoporotic fracture risk. The previous study showed that age was one of the most important risk factors for the development of fragility fractures. Prevention is the key to master their management, including the use of drugs against osteoporosis [22]. In terms of treatment, evidence from a Bayesian network meta-analysis demonstrated that alendronate, risedronate, zoledronate and denosumab were effective in increasing bone density in the spine and reducing vertebral fractures in patients taking corticosteroids. Alendronate, zoledronate and denosumab increased BMD in the hip. Alendronate produced increased femoral neck and hip BMDs, reduced incidence of novel fractures [23]. Another network meta-analysis showed that denosumab followed by pamidronate and zoledronate was associated with higher spine BMD in selected women with postmenopausal osteoporosis. Denosumab followed by alendronate and ibandronate had the highest influence on hip and femoral BMD. Future studies should evaluate the effects of anti-osteoporosis drugs on the overall fracture risk and consider other types of osteoporosis [24]. Moreover, the present analysis supported the adoption of bone turnover (BMTs) during pharmacological therapy setting and therapy monitoring of patients suffering from osteoporosis [25, 26]. A Bayesian network meta-analysis of RCTs demonstrated that denosumab resulted in most effective in preventing osteoporotic fractures, particularly in reducing the occurrence of nonvertebral fractures. Romosozumab and ibandronate, on the other hand, provided the best evidence for preventing vertebral fractures and hip fractures, respectively [27].

Therefore, it is essential to gain a comprehensive understanding of fracture predictors in postmenopausal women which was essential to effectively plan treatment and preventive strategies, but there was still a scarcity of relevant review studies. Existing studies exploring predictors of osteoporotic fractures in postmenopausal women often suffer from limitations, such as small sample sizes and potential publication bias. To optimizing preventive strategies, there is a pressing need for further research focused on identifying easily accessible and specific predictors of fractures in postmenopausal women. In our study, we aimed to address these gaps by employing broader inclusion criteria and comprehensive search strategies, allowing for a more thorough exploration of the predictors of fractures in postmenopausal women. By doing so, we hope to contribute valuable insights that can inform and enhance preventive measures in this vulnerable population.

## Methods

### Data sources and searches

A prospective protocol was used to comply with the Preferred Reporting Items for Systematic Reviews and Meta-Analyses (PRISMA) guidelines. The PROSPERO ID of this Systematic Review’s protocol was CRD42022355407. We have searched Embase, MEDLINE and Cochrane with search terms (postmenopausal AND fracture) AND (“risk factor” OR “predictive factor”) in May 2022. Additionally, the references of the included literatures and previous reviews of fractures in postmenopausal women were screened. The related articles in the references were included.

### Inclusion and exclusion criteria

The inclusion criteria were as follows:

(i) Cohort or case–control studies were conducted on postmenopausal women with fractures and published full-text reports in peer-reviewed journals in English;
(ii) Availability of detailed reports on postmenopausal women in the study;
(iii) Postmenopausal was defined as the absence of menstruation for a minimum of 1 year [28].

Our calculations and analysis were based on the raw data provided by the included studies. The articles without clinical information were excluded. Likewise, we also excluded experiments on animals, reviews, case reports, expert opinions, editorials and correspondence.

### Quality assessment and data extraction

The Newcastle–Ottawa Scale (NOS) was used to assess three aspects of the included studies: basis of case selection, comparability of the study groups and outcome assessment [29]. The quality assessment was conducted by two independent reviewers who assigned stars based on the prespecified criteria. Bias was determined to be low in studies that scored four stars for selection, two stars for comparability and three stars for determining the outcome. On the NOS, studies with at least seven stars were considered to be of high quality [30]. A predefined data extraction form was used by two investigators to extract data independently and systematically. When disagreements could not be resolved through consensus, a third senior investigator was referred.

### Data analysis

Statistical analyses were conducted in Stata 14.0 and RevMan statistical software. To analyze the raw data, at least two studies had to be conducted on the same potential predictor. For dichotomous outcomes, odds ratios (ORs) and 95% confidence intervals (CIs) were calculated, and mean differences (MDs) with 95% CIs were calculated for continuous outcomes. In cases where the *P* value for dichotomous outcomes was significant (*P* < 0.05), the sensitivity and specificity of the model were analyzed. According to the methods proposed by Luo et al. or Wan et al., we converted raw data, such as medians, ranges or quartiles, into means and standard deviations (SD) when the data means and/or standard deviations were not provided in the included studies [31, 32]. We combined data from cohort studies and case–control studies.

Cochran’s Q statistic and the *I*^2^ statistic were used to investigate heterogeneity. According to *I*^2^ statistics of 25%, 50% or 75%, heterogeneity was classified as low, medium or high, respectively [33]. Additionally, we examined potential explanations for heterogeneity through sensitivity analyses. Moreover, since the fracture sites were different in the included studies (hip, wrist and spine), which may affect the analysis results, we conducted the subgroup analyses. When sensitivity analysis or subgroup analysis could not identify the source of heterogeneity, random-effect models were used instead of fixed-effect models. Publication bias was assessed using Begg’s and Egger’s tests. *P* < 0.05 was considered statistically significant. If there was significant publication bias, an estimate from trim-and-fill analysis was reported.

## Results

### Literature search

**Fig. 1:**
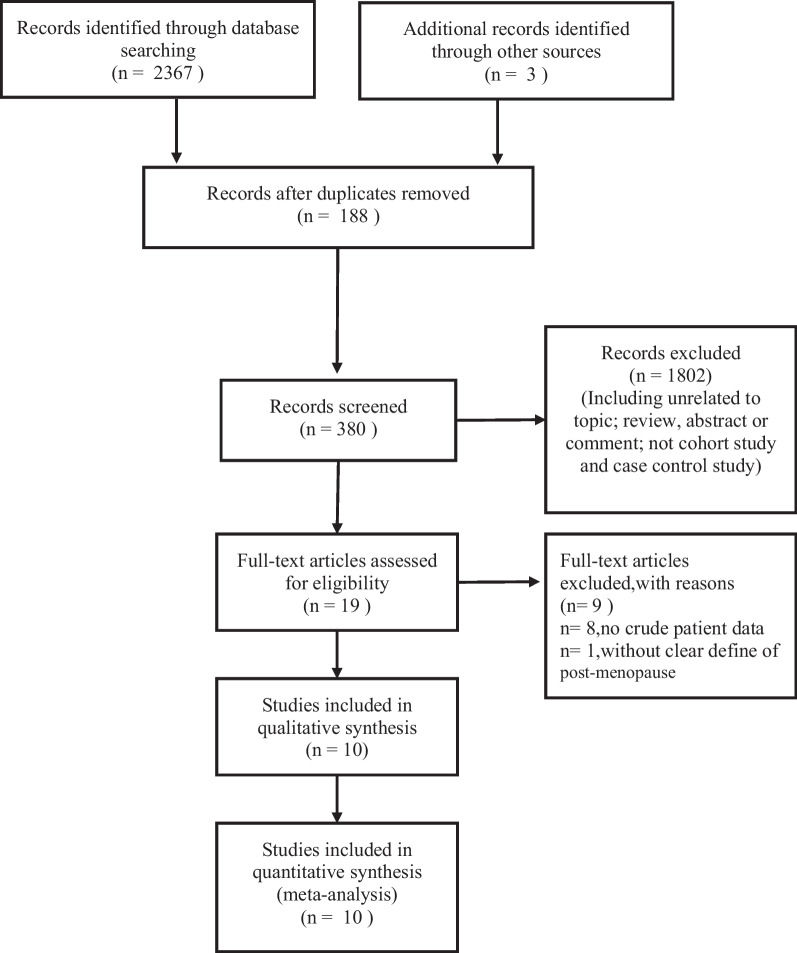
Process of searching for studies and screening

Literature search and study selection are shown in Fig. 1. A total of 2370 citations were identified, and 19 abstracts were selected for detailed evaluation. Nine studies were excluded after meticulously reviewing the full texts. Finally, 10 studies with 1,287,021 postmenopausal women were found eligible for analyses [13, 34–42]. In the eligible studies, the sample size ranged from 311 to 1,272,115. The years of the surveys ranged from 1993 to 2021.

### Study characteristics

A summary of the baseline characteristics of the studies is shown in Table 1. A total of 10 studies were included (six cohort studies and four case–control studies). Four studies provided predictors of hip fractures in postmenopausal women. One study compared predictors of incident vertebral fracture and non-incident vertebral fracture. There were five studies with unspecified fractures types. According to NOS assessment, five of the 10 studies (50%) were found to have medium or high risk of bias.

**Table 1:** Baseline characteristics of the studies included in the meta-analysis

Study	Year	Study design	Total number	Persons with fractures	Age at inclusion	Nation	Fracture site	Study quality (star rating)
ROBERT G CUMMING	1993	Case–control	311	137	≥ 65	Sydney, Australia	Hip fractures	********
Fabio Parazzini	1996	Case–control	796	206	55–74	Milan, Italy	Hip fractures	******
Alexandra Papaioannou	2005	Cohort	5143	314	NA	Canadian	Any type of fracture	*******
MARJO TUPPURAINEN	1995	Cohort	3140	157	47–56	Kuopio Province, Eastern Finland	NA	******
Dezheng Huo	2003	Case–control	354	118	≥ 50	Beijing	Hip fracture	******
Marjolein van der Klift	2004	Cohort	1624	113	≥ 55	Rotterdam	Vertebral fracture	********
Marjorie R. Jenkins	2008	Case–control	488	190	≥ 50	Northwest Texas	Hip fracture	*****
Florence A Tre´mollieres	2010	Cohort	2196	145	≥ 45	Toulouse, France	Any type of fracture	********
Jung Eun Yoo	2021	Cohort	1,272,115	189,883	≥ 40	Korean	Any type of fracture	********
Rafaela Martinez Copês	2021	Cohort	854	58	≥ 55	Southern Brazil	NA	******

### Meta-analysis

In the 10 articles, mean age of patients ranged from 54.8 to 77.9 years old, and 14.87% (191,321/1,287,021) of postmenopausal women developed fracture. With these 10 articles, we identified a total of 15 potential predictors of fracture. Among them, 12 predictors were statistically significant (*P* < 0.05), and nine predictors were highly correlated with fracture in postmenopausal women (*P* < 0.01) (Table 2). Statistically significant differences were observed in age, BMI, senior high school and above, parity ≥ 3, history of hypertension, history of diabetes mellitus, history of alcohol intake, age at menarche ≥ 15, age at menopause < 40, age at menopause > 50, estrogen use and vitamin D supplements. The forest plots are shown in Additional file 1.

**Table 2:** Results of pooled analysis, sensitivity analysis and subgroup analysis performed for the studies included

Predictor	Estimates (95% CI)	*P* value for overall effect	*P* value for heterogeneity	*P* value for Begg’s test	*P* value for Egger’s test	*I*^2^ (%)	Sensitivity	Specificity
*Meta-analysis*								
Age	MD 1.93 (0.61, 3.26)	0.004	< 0.00001	0.710	0.998	97		
BMI	MD -0.69 (− 1.31, − 0.07)	0.03	< 0.00001	0.198	0.387	90	0.43 (0.36, 0.51)	0.59 (0.51, 0.66)
Senior high and above	1.76 (1.34, 2.32)	< 0.0001	0.85			0	0.44 (0.34, 0.54)	0.70 (0.62, 0.76)
Parity ≥ 3	0.74 (0.58, 0.94)	0.01	0.41			0		
History of hypertension	1.20 (1.19, 1.22)	< 0.00001	0.45			0	0.38 (0.33, 0.42)	0.69 (0.61, 0.76)
History of diabetes mellitus	1.19 (1.17, 1.20)	< 0.00001	0.65			0	0.07 (0.04, 0.10)	0.93 (0.92, 0.94)
History of alcohol intake	0.89 (0.88, 0.90)	< 0.00001	0.0004			87	0.27 (0.15, 0.45)	0.78 (0.63, 0.88)
Smoking	1.16 (0.91, 1.48)	0.23	< 0.0001	1.000	0.005	81		
Age at menarche < 12	1.22 (0.91, 1.63)	0.18	0.40			0		
Age at menarche ≥ 15	1.34 (1.03, 1.73)	0.03	0.24			30	0.24 (0.20, 0.28)	0.83 (0.81, 0.85)
Age at menopause < 40	1.23 (1.19, 1.28)	< 0.00001	0.18			42	0.17 (0.12, 0.24)	0.86 (0.74, 0.93)
Age at menopause > 50	0.96 (0.95, 0.97)	< 0.00001	0.64			0	0.33 (0.25, 0.42)	0.65 (0.53, 0.75)
Estrogen use	0.53 (0.28, 0.87)	< 0.00001	0.46	0.452	0.199	0	0.18 (0.09, 0.34)	0.71 (0.50, 0.86)
Calcium daily intake (mg)	3.61 (− 37.42, 44.64)	0.86	0.29			19		
Vitamin D supplements	1.75 (1.35, 2.28)	< 0.0001	0.17			47	0.25 (0.04, 0.72)	0.81 (0.46, 0.96)
*Sensitivity analysis*								
BMI	MD -0.29 (− 0.63, − 0.06)	0.10	0.02			61		
History of alcohol intake	0.89 (0.88, 0.91)	< 0.0001	0.47			0		
*Subgroup analysis*								
Smoking	1.16 (0.91, 1.48)	0.23	< 0.0001			81		
Hip fractures	1.76 (1.20, 2.58)	0.004	0.13			56		
Fractures of any location	1.01 (0.83, 1.21)	0.095	0.07			54		

Among the 15 predictors of fracture in postmenopausal women, four factors were with significant heterogeneity (*I*^2^ > 50%). As a result, sensitivity analysis was conducted for these factors. Two predictors (BMI and history of alcohol intake) had an *I*^2^ of 0–61% after one low-quality article was removed (see Table 2). We divided the four predictors with significant heterogeneity into two subgroups based on fracture type using subgroup analysis. Subgroup analysis showed that smoking (OR = 1.76, 95% CI 1.20–2.58) was correlated with fractures in hip fractures subgroup (*P* < 0.05) (Table 2). Moreover, given predictor of age could not be found the source of heterogeneity by subgroup analysis or sensitivity analysis, we used random-effect models instead of fixed-effect models for meta-analysis to increase the reliability. There was no difference between the results of the two models. Additionally, there were four predictors which were researched in more than 5 articles (Table 2). These predictors were analyzed for publication bias using Egger’s and Begg’s tests. Smoking was found to have publication bias (see Table 2). However, the trim-and-fill analysis of this factor showed no significant logarithmic risk ratios, which indicated no publication bias.

## Discussion

Osteoporosis and osteoporosis-related fractures are more prevalent among postmenopausal women compared to premenopausal women or men, primarily due to the rapid bone loss accompanied by the decline of ovarian function in the menopausal transition. Our results suggested that several factors were associated with osteoporotic fractures in postmenopausal women, including age, BMI, senior high school and above, parity ≥ 3, history of hypertension, history of diabetes mellitus, history of alcohol intake, age at menarche ≥ 15, age at menopause < 40, age at menopause > 50, estrogen use and vitamin D supplements.

The decline in estrogen levels following menopause leads to reduced bone deposition, particularly in weight-bearing bones, while also increasing bone resorption [43]. Endogenous estrogen exposure mainly occurs during the reproductive phase, encompassing the time between menarche and menopause. Menopause age < 40 and menarche age ≥ 15 indicated the shorter reproductive period, while the menopause age > 50 represented longer reproductive period. The shorter the reproductive period, the less exposure to estrogen. It could explain that earlier menopause (menopause age < 40), later menarche (menarche age ≥ 15) and shorter reproductive span were associated with higher fracture risk in postmenopausal women. Menopause age > 50 was possibly a protective factor. Moreover, the endogenous estrogen exposure was negatively correlated with the risk of all fracture sites combined, as well as hip and vertebral fractures specifically. Therefore, our study confirmed the association between lower lifetime endogenous estrogen exposure and increased fracture incidence, which was revealed by the previous literature [13].

It has been reported that parous women have a lower risk of fractures compared to nulliparous women [13]. Pregnancy was closely associated with women’s metabolic changes through great influence on their BMD [44]. When mother’s intestinal calcium absorption was inadequate to meet calcium demand to support fetal skeletal growth during pregnancy, the fetal system compensates by obtaining calcium from the mother’s skeleton [45–47]. This may increase the long-term fracture risk of the mothers by reducing bone mass. Meanwhile, the socioeconomic condition and lifestyle factors during pregnancy may also play a crucial role in the fracture risk [48]. On the other side, increased bone loading and higher serum estrogen levels during pregnancy may protect against maternal bone loss. A meta-analysis of 10 prospective studies, including 217,295 participants (26,525 osteoporotic fracture patients), demonstrated an inverse dose–response association between parity and the risk of osteoporotic fracture and hip fracture [44].

The mechanism underlying the association between parity and osteoporotic fracture and hip fracture risks among postmenopausal women may be explained by some potential biological mechanisms. During pregnancy, serum estrogen levels rose to about 20–30 times levels above their normal menstrual cycles’ peak. Such heightened endogenic estrogen exposure during pregnancy may reduce the fracture risk. Some nulliparous women could be at a greater risk of fracture due to the subfertility, which produced less endogenic estrogen during their normal menstrual cycle compared to more fertile women [49]. The most likely mechanism by which parity protected women against hip fracture was through increased bone formation rates during pregnancy, resulting in the increases in bone mass [50].

Many changes took place in hip and pelvic alignment during pregnancy and childbirth to alter hip structure permanently, which could, in turn, prevent women from future fracture. Moreover, since estrogens appeared to improve neuromuscular performance and muscle strength, it may also protect against hip fracture by reducing injurious falls. As an exogenous hormonal exposure, the previous study also reported that the hormone therapy (HT) was independently associated with a lower fracture risk in postmenopausal women [51]. Benefits of postmenopausal estrogen therapy have been proven in reducing the fracture risk, including risk of total, vertebral and hip fractures. It is not clearly known about the mechanisms underlying the association between HT and the lower fracture risk so far. It is hypothesized that HT improved calcium retention through increased renal calcium reabsorption and intestinal calcium absorption [52]. The mechanism was also thought to involve the osteoclasts inhibition, leading to decreased bone turnover and improved the balance between bone resorption and formation [53, 54]. Our results provided further support to the hypothesis that exogenous female HT may prevent fracture through the beneficial effects of estrogen on bone metabolism.

Physical activity has been demonstrated to increase BMD and muscle strength, which have the effects of improved muscle balance, control and coordination and reduced fall risks, especially in the elderly [55]. Active rehabilitation may be one of the most critical factors for the prevention of future fracture risk due to low BMD, in the form of structured exercise [56, 57]. The structured exercise program also contributed to improve the quality of life in the postmenopausal women with low BMD. Several RCT researches have reported the positive effects of weight-bearing activities to reduce risks of fall and fracture through increasing BMD in postmenopausal women with low BMD by improving muscle strength and physical function [58–60].

Those people with higher levels of education may not have enough time for exercise. This could explain that the education level was inversely related to fracture risk in postmenopausal women. However, the association between education degree and the fracture risk remained controversial. Shaw et al. found no significant associations between BMD and education degree in a cross-sectional study in Taiwan [61]. On the contrary, Ho et al. demonstrated that a higher education level was associated with improved BMD and a lower osteoporosis prevalence among Chinese postmenopausal women [62]. The similar conclusion was drawn in a Taiwan population [48]. Based on the association between low socioeconomic status and increased incidence of hip fracture, the researchers regarded the lower level of education as a risk factor of first-incident hip fracture [63]. Colon Emeric et al. [64] observed a positive association between the educational level and the hip fracture risk among ambulatory non-Hispanic White men, which supported the conclusions of our study. Postmenopausal women with a low education degree predominated in nearly all groups, what may have potentially decreased the multivariate analysis power on this variable. The inconsistent conclusions may be attributed to the ethnic, culture difference between Eastern and Western countries, as well as to the different research types, sampling methods and limited sample sizes.

Chen et al. also reported that steroid use and diabetes mellitus increased the risk of first-incident hip fracture. It was generally acknowledged that common chronic diseases linked with increased risk of falls in the elderly, such as diabetes mellitus and hypertension [48]. Type 2 diabetes mellitus (T2DM) was associated with the increased fracture risk, which resulted in increased risk of mortality and disability in women [65]. Similarly, premenopausal women with type 1 diabetes (T1DM) also needed to take precautions for osteoporosis [66]. Large cohort studies have shown that women with diabetes had twice the risk of hip fracture compared with those without diabetes after controlling for the confounding effect of areal BMD, which was measured by dual-energy X-ray absorptiometry (DXA). The previous study demonstrated that elevated homeostasis model assessment of IR (HOMA-IR) was associated with lower bone strength indices and cortical bone volume in nondiabetic postmenopausal women, independent of age and body size [67]. Exact mechanisms of hyperinsulinemia, possible differences in insulin sensitivity and impaired insulin signaling among bone cells or other organs were not fully revealed yet. Postulated pathophysiological mechanisms included increased formation of advanced glycation end-products (AGEs) in the bone and impaired bone microvasculature [68]. Recent large population-based study revealed that sulfonylurea was associated with an adjusted hazard ratio (HR) of 1.3 for major osteoporotic fracture events [69]. Another study also suggested that higher level of serum sex hormone-binding globulin (SHBG) was associated with lower BMDs, higher osteopenia/osteoporosis risk and future fracture risk calculated by FRAX [70]. It was widely accepted that SHBG involved in bone metabolism through the anti-estrogenic effect. As a transport protein, higher SHBG binds to estrogen main sex hormones including circulating E2 and T, transporting them toward target cells and reducing its biologically active form, which consequently reduces BMD and increases future fracture risk [71]. Moreover, decreased levels of bone quality were associated with an increased risk of fracture in old women with diabetes [72]. In postmenopausal women with type 2 diabetes, some studies on bone microstructure have shown that cortical bone density decreases obviously, which accounted for 90% of bone composition and played a key role in bone weight-bearing and anti-traumatic activities [67]. Other contributing factors consisted of an accumulation of the development of diabetes complications (such as hypoglycemia and neuropathy) and advanced glycation end-products, which led to further drop of BMD, worsening geometric properties within bone and increased risk of fracture and fall [73].

Vitamin D use may indicate a prevention strategy, which was associated with an increased risk of any nonvertebral fracture [38]. It is likely that more individuals may take vitamin D if they have a deficiency, leading to the association between vitamin D use and increased fracture risk. Our analysis also showed that vitamin D use is a risk factor of osteoporotic fracture in postmenopausal women. Two important UK studies have not shown positive effect of vitamin D and calcium supplementation on the free-living elderly women’s fracture prevention [74, 75]. This discrepancy in findings highlights the complexity of the relationship between vitamin D use and fracture risk, and further research is needed to better understand this association.

This study has several limitations. First, since it was hard to separately analyze the effects of possible interventional treatments on osteoporotic fracture in postmenopausal women, the potential effect of interventional treatments on the predictive factors remained unknown. Larger prospective with a more substantial sample size studies is needed to validate and corroborate our results. Secondly, some of the identified predictors may act as possible covariates, with part of them are independent predictors. The current methodology is unable to identify the independent predictors of osteoporotic fracture in postmenopausal women. Thirdly, it is difficult to show causality in cohort and case–control studies, and thus, the results primarily represent associations rather than causal relationships.

In conclusion, our meta-analysis of 10 articles has successfully identified the most relevant predictors of osteoporotic fracture in postmenopausal women. These findings will facilitate the early screening and identification of high-risk individuals, thus enabling timely preventive and therapeutic interventions. For better evaluation of the risk of osteoporotic fracture in postmenopausal women, future larger sample prospective studies are needed to confirm our major findings.

### Supplementary Information

**Additional file 1.** Forest plots of the main subgroups in the study.

## Data Availability

The datasets used and/or analyzed during the current study are available from the corresponding author on reasonable request.

## References

[1] Newgard CB, Sharpless NE (2013). Coming of age: molecular drivers of aging and therapeutic opportunities. J Clin Invest.

[2] Srivastava M, Deal C (2002). Osteoporosis in elderly: prevention and treatment. Clin Geriatr Med.

[3] Haentjens P, Magaziner J, Colon-Emeric CS, Vanderschueren D, Milisen K, Velkeniers B, Boonen S (2010). Meta-analysis: excess mortality after hip fracture among older women and men. Ann Intern Med.

[4] Clynes MA, Harvey NC, Curtis EM, Fuggle NR, Dennison EM, Cooper C (2020). The epidemiology of osteoporosis. Br Med Bull.

[5] Wang L, Yu W, Yin X, Cui L, Tang S, Jiang N, Cui L, Zhao N (2021). Prevalence of osteoporosis and fracture in China: the China osteoporosis prevalence study. JAMA Netw Open.

[6] Kanis JA, Melton LJ, Christiansen C, Johnston CC, Khaltaev N (1994). The diagnosis of osteoporosis. J Bone Miner Res.

[7] Compston JE, McClung MR, Leslie WD (2019). Osteoporosis. Lancet.

[8] Kanis JA (2002). Diagnosis of osteoporosis and assessment of fracture risk. Lancet.

[9] Kanis JA, Svedbom A, Harvey N, McCloskey EV (2014). The osteoporosis treatment gap. J Bone Miner Res.

[10] Bae G, Kim E, Kwon HY, Ha YC, An J, Park J, Yang H (2020). Burden of osteoporotic fractures using disability-adjusted life years in South Korea. Asia Pac J Public Health.

[11] Kang HY, Yang KH, Kim YN, Moon SH, Choi WJ, Kang DR, Park SE (2010). Incidence and mortality of hip fracture among the elderly population in South Korea: a population-based study using the national health insurance claims data. BMC Public Health.

[12] Lin J, Zhu J, Wang Y, Zhang N, Gober HJ, Qiu X, Li D, Wang L (2017). Chinese single herbs and active ingredients for postmenopausal osteoporosis: from preclinical evidence to action mechanism. Biosci Trends.

[13] Yoo JE, Shin DE, Han K, Kim D, Yoon JW, Lee DY (2021). Association of female reproductive factors with incidence of fracture among postmenopausal women in Korea. JAMA Netw Open.

[14] Khosla S, Oursler MJ, Monroe DG (2012). Estrogen and the skeleton. Trends Endocrinol Metab.

[15] Nelson HD (2003). Postmenopausal osteoporosis and estrogen. Am Fam Physician.

[16] Zhang Q, Greenbaum J, Zhang WD, Sun CQ, Deng HW (2018). Age at menarche and osteoporosis: a Mendelian randomization study. Bone.

[17] Johnell O, Gullberg B, Kanis JA, Allander E, Elffors L, Dequeker J, Dilsen G, Gennari C (1995). Risk factors for hip fracture in European women: the MEDOS study Mediterranean osteoporosis study. J Bone Miner Res.

[18] Shimizu Y, Sawada N, Nakamura K, Watanabe Y, Kitamura K, Iwasaki M, Tsugane S (2018). Menstrual and reproductive factors and risk of vertebral fractures in Japanese women: the Japan Public Health Center-based prospective (JPHC) study. Osteoporos Int.

[19] Peng K, Yao P, Kartsonaki C, Yang L, Bennett D, Tian M, Li L, Guo Y (2020). Menopause and risk of hip fracture in middle-aged Chinese women: a 10-year follow-up of China Kadoorie Biobank. Menopause.

[20] Svejme O, Ahlborg HG, Nilsson JA, Karlsson MK (2012). Early menopause and risk of osteoporosis, fracture and mortality: a 34-year prospective observational study in 390 women. BJOG.

[21] Sullivan SD, Lehman A, Thomas F, Johnson KC, Jackson R, Wactawski-Wende J, Ko M, Chen Z (2015). Effects of self-reported age at nonsurgical menopause on time to first fracture and bone mineral density in the Women's Health Initiative Observational Study. Menopause.

[22] Migliorini F, Giorgino R, Hildebrand F, Spiezia F, Peretti GM, Alessandri-Bonetti M, Eschweiler J, Maffulli N (2021). Fragility fractures: risk factors and management in the elderly. Medicina.

[23] Migliorini F, Colarossi G, Eschweiler J, Oliva F, Driessen A, Maffulli N (2022). Antiresorptive treatments for corticosteroid-induced osteoporosis: a Bayesian network meta-analysis. Br Med Bull.

[24] Migliorini F, Maffulli N, Colarossi G, Eschweiler J, Tingart M, Betsch M (2021). Effect of drugs on bone mineral density in postmenopausal osteoporosis: a Bayesian network meta-analysis. J Orthop Surg Res.

[25] Migliorini F, Maffulli N, Spiezia F, Peretti GM, Tingart M, Giorgino R (2021). Potential of biomarkers during pharmacological therapy setting for postmenopausal osteoporosis: a systematic review. J Orthop Surg Res.

[26] Migliorini F, Maffulli N, Spiezia F, Tingart M, Maria PG, Riccardo G (2021). Biomarkers as therapy monitoring for postmenopausal osteoporosis: a systematic review. J Orthop Surg Res.

[27] Migliorini F, Colarossi G, Baroncini A, Eschweiler J, Tingart M, Maffulli N (2021). Pharmacological management of postmenopausal osteoporosis: a level I evidence based - expert opinion. Expert Rev Clin Pharmacol.

[28] Gracia CR, Sammel MD, Freeman EW, Lin H, Langan E, Kapoor S, Nelson DB (2005). Defining menopause status: creation of a new definition to identify the early changes of the menopausal transition. Menopause.

[29] The Newcastle-Ottawa Scale (NOS) for assessing the quality of nonrandomized studies in meta-analysis. Available: www.ohri.ca/programs/clinical_epidemiology/oxford.asp. Accessed 25 Nov 2012.

[30] Viale L, Allotey J, Cheong-See F, Arroyo-Manzano D, Mccorry D, Bagary M, Mignini L, Khan KS (2015). Epilepsy in pregnancy and reproductive outcomes: a systematic review and meta-analysis. Lancet.

[31] Luo D, Wan X, Liu J, Tong T (2018). Optimally estimating the sample mean from the sample size, median, mid-range, and/or mid-quartile range. Stat Methods Med Res.

[32] Wan X, Wang W, Liu J, Tong T (2014). Estimating the sample mean and standard deviation from the sample size, median, range and/or interquartile range. BMC Med Res Methodol.

[33] Higgins JP, Thompson SG, Deeks JJ, Altman DG (2003). Measuring inconsistency in meta-analyses. BMJ.

[34] Cumming RG, Klineberg RJ (1993). Breastfeeding and other reproductive factors and the risk of hip fractures in elderly women. Int J Epidemiol.

[35] Parazzini F, Tavani A, Ricci E, La Vecchia C (1996). Menstrual and reproductive factors and hip fractures in post menopausal women. Maturitas.

[36] Papaioannou A, Joseph L, Ioannidis G, Berger C, Anastassiades T, Brown JP, Hanley DA, Hopman W (2005). Risk factors associated with incident clinical vertebral and nonvertebral fractures in postmenopausal women: the Canadian Multicentre Osteoporosis Study (CaMos). Osteoporos Int.

[37] Tuppurainen M, Kroger H, Honkanen R, Puntila E, Huopio J, Saarikoski S, Alhava E (1995). Risks of perimenopausal fractures–a prospective population-based study. Acta Obstet Gynecol Scand.

[38] Huo D, Lauderdale DS, Li L (2003). Influence of reproductive factors on hip fracture risk in Chinese women. Osteoporos Int.

[39] van der Klift M, de Laet CE, McCloskey EV, Johnell O, Kanis JA, Hofman A, Pols HA (2004). Risk factors for incident vertebral fractures in men and women: the Rotterdam Study. J Bone Miner Res.

[40] Jenkins MR, Denison AV (2008). Smoking status as a predictor of hip fracture risk in postmenopausal women of northwest Texas. Prev Chronic Dis.

[41] Tremollieres FA, Pouilles JM, Drewniak N, Laparra J, Ribot CA, Dargent-Molina P (2010). Fracture risk prediction using BMD and clinical risk factors in early postmenopausal women: sensitivity of the WHO FRAX tool. J Bone Miner Res.

[42] Copes RM, Comim FV, Barrios NS, Premaor MO (2021). Incidence of fractures in women in the post-menopause: a cohort study in primary care in southern Brazil. Arch Osteoporos.

[43] Raisz LG (2005). Pathogenesis of osteoporosis: concepts, conflicts, and prospects. J Clin Invest.

[44] Wang Q, Huang Q, Zeng Y, Liang JJ, Liu SY, Gu X, Liu JA (2016). Parity and osteoporotic fracture risk in postmenopausal women: a dose-response meta-analysis of prospective studies. Osteoporos Int.

[45] Black AJ, Topping J, Durham B, Farquharson RG, Fraser WD (2000). A detailed assessment of alterations in bone turnover, calcium homeostasis, and bone density in normal pregnancy. J Bone Miner Res.

[46] Kovacs CS (2001). Calcium and bone metabolism in pregnancy and lactation. J Clin Endocrinol Metab.

[47] Prentice A (2000). Calcium in pregnancy and lactation. Annu Rev Nutr.

[48] Chen FP, Fu TS, Lin YC, Fan CM (2018). Risk factors and quality of life for the occurrence of hip fracture in postmenopausal women. Biomed J.

[49] Hillier TA, Rizzo JH, Pedula KL, Stone KL, Cauley JA, Bauer DC, Cummings SR (2003). Nulliparity and fracture risk in older women: the study of osteoporotic fractures. J Bone Miner Res.

[50] Bjornerem A, Ahmed LA, Jorgensen L, Stormer J, Joakimsen RM (2011). Breastfeeding protects against hip fracture in postmenopausal women: the Tromso study. J Bone Miner Res.

[51] Rossouw JE, Anderson GL, Prentice RL, LaCroix AZ, Kooperberg C, Stefanick ML, Jackson RD, Beresford SA (2002). Risks and benefits of estrogen plus progestin in healthy postmenopausal women: principal results from the Women's Health Initiative randomized controlled trial. JAMA.

[52] Michaelsson K, Baron JA, Farahmand BY, Johnell O, Magnusson C, Persson PG, Persson I, Ljunghall S (1998). Hormone replacement therapy and risk of hip fracture: population based case-control study. The Swedish Hip Fracture Study Group. BMJ.

[53] Hillard TC, Stevenson JC (1991). Role of oestrogen in the development of osteoporosis. Calcif Tissue Int.

[54] Rizzoli R, Bonjour JP (1997). Hormones and bones. Lancet.

[55] Ashe MC, Santos IKD, Edward NY, Burnett LA, Barnes R, Fleig L, Puyat JH, Sale JEM (2021). Physical activity and bone health in men: a systematic review and meta-analysis. J Bone Metab.

[56] Howe TE, Shea B, Dawson LJ, Downie F, Murray A, Ross C, Harbour RT, Caldwell LM (2011). Exercise for preventing and treating osteoporosis in postmenopausal women. Cochrane Database Syst Rev.

[57] Karlsson MK, Nordqvist A, Karlsson C (2008). Physical activity, muscle function, falls and fractures. Food Nutr Res.

[58] de Kam D, Smulders E, Weerdesteyn V, Smits-Engelsman BC (2009). Exercise interventions to reduce fall-related fractures and their risk factors in individuals with low bone density: a systematic review of randomized controlled trials. Osteoporos Int.

[59] Gillespie LD, Gillespie WJ, Robertson MC, Lamb SE, Cumming RG, Rowe BH (2003). Interventions for preventing falls in elderly people. Cochrane Database Syst Rev.

[60] Howe TE, Rochester L, Neil F, Skelton DA, Ballinger C (2011). Exercise for improving balance in older people. Cochrane Database Syst Rev.

[61] Shaw CK (1993). An epidemiologic study of osteoporosis in Taiwan. Ann Epidemiol.

[62] Ho SC, Chen YM, Woo JL (2005). Educational level and osteoporosis risk in postmenopausal Chinese women. Am J Epidemiol.

[63] Quah C, Boulton C, Moran C (2011). The influence of socioeconomic status on the incidence, outcome and mortality of fractures of the hip. J Bone Jt Surg Br.

[64] Colon-Emeric CS, Biggs DP, Schenck AP, Lyles KW (2003). Risk factors for hip fracture in skilled nursing facilities: who should be evaluated?. Osteoporos Int.

[65] de Castro GD, Valadares AL, Pinto-Neto AM, Morais SS, Costa-Paiva L (2012). Ability to follow drug treatment with calcium and vitamin D in postmenopausal women with reduced bone mass. Menopause.

[66] Russo GT, Giandalia A, Romeo EL, Nunziata M, Muscianisi M, Ruffo MC, Catalano A, Cucinotta D (2016). Fracture risk in type 2 diabetes: current perspectives and gender differences. Int J Endocrinol.

[67] Yoshioka F, Nirengi S, Murata T, Kawaguchi Y, Watanabe T, Saeki K, Yoshioka M, Sakane N (2021). Lower bone mineral density and higher bone resorption marker levels in premenopausal women with type 1 diabetes in Japan. J Diabetes Investig.

[68] Yang J, Hong N, Shim JS, Rhee Y, Kim HC (2018). Association of insulin resistance with lower bone volume and strength index of the proximal femur in nondiabetic postmenopausal women. J Bone Metab.

[69] Lafage-Proust MH, Roche B, Langer M, Cleret D, Vanden Bossche A, Olivier T, Vico L (2015). Assessment of bone vascularization and its role in bone remodeling. Bonekey Rep.

[70] Majumdar SR, Josse RG, Lin M, Eurich DT (2016). Does sitagliptin affect the rate of osteoporotic fractures in type 2 diabetes? Population-Based Cohort Study. J Clin Endocrinol Metab.

[71] Jing Y, Wang X, Yu J, Wang X, Zhou Y, Tao B, Sun L, Liu J (2019). Associations of serum sex hormone binding globulin with bone mineral densities and higher 10-year probability of fractures in postmenopausal women with type 2 diabetes mellitus. Ann Transl Med.

[72] Legrand E, Hedde C, Gallois Y, Degasne I, Boux De Casson F, Mathieu E, Basle MF, Chappard D (2001). Osteoporosis in men: a potential role for the sex hormone binding globulin. Bone.

[73] Li J, Niu X, Si Q, Song Q, Jin M, Zhou R, Sun Y, Li J (2021). Plasma periostin as a biomarker of osteoporosis in postmenopausal women with type 2 diabetes. J Bone Miner Metab.

[74] Porthouse J, Cockayne S, King C, Saxon L, Steele E, Aspray T, Baverstock M, Birks Y (2005). Randomised controlled trial of calcium and supplementation with cholecalciferol (vitamin D3) for prevention of fractures in primary care. BMJ.

[75] Grant AM, Avenell A, Campbell MK, McDonald AM, MacLennan GS, McPherson GC, Anderson FH, Cooper C (2005). Oral vitamin D3 and calcium for secondary prevention of low-trauma fractures in elderly people (Randomised Evaluation of Calcium or vitamin D, RECORD): a randomised placebo-controlled trial. Lancet.

