# Supplementary figures and images for: Predictors of osteoporotic fracture in postmenopausal women: a meta-analysis
Source: J Orthop Surg Res. 2023 Aug 5;18:574. doi: 10.1186/s13018-023-04051-6 (PMC10404374; doi:10.1186/s13018-023-04051-6)

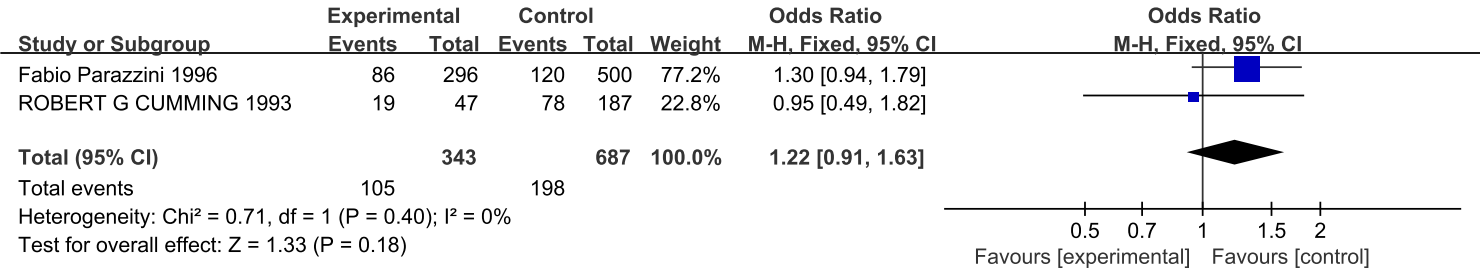

Supplement: Supplementary file 1 — Additional file 1. Forest plots of the main subgroups in the study. [file 13018_2023_4051_MOESM1_ESM.zip › Supplementary material/Age at menarche<12—Forest plot.pdf]

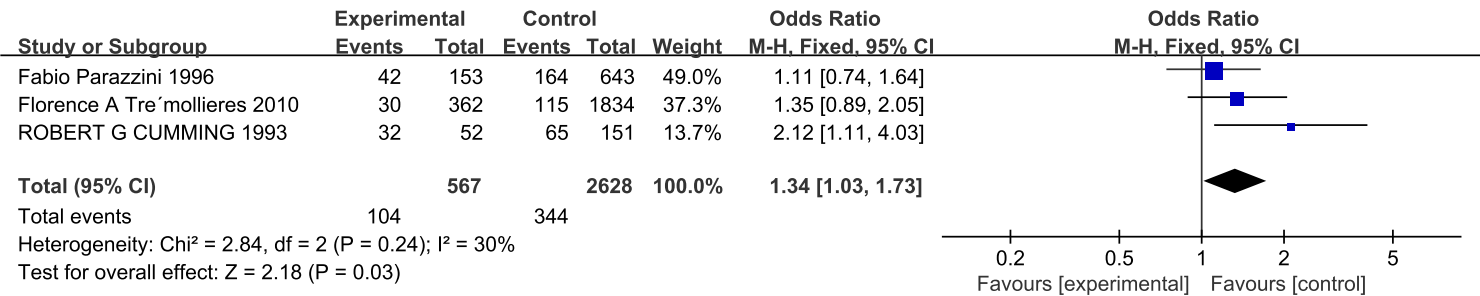

Supplement: Supplementary file 1 — Additional file 1. Forest plots of the main subgroups in the study. [file 13018_2023_4051_MOESM1_ESM.zip › Supplementary material/Age at menarche>15—Forest plot.pdf]

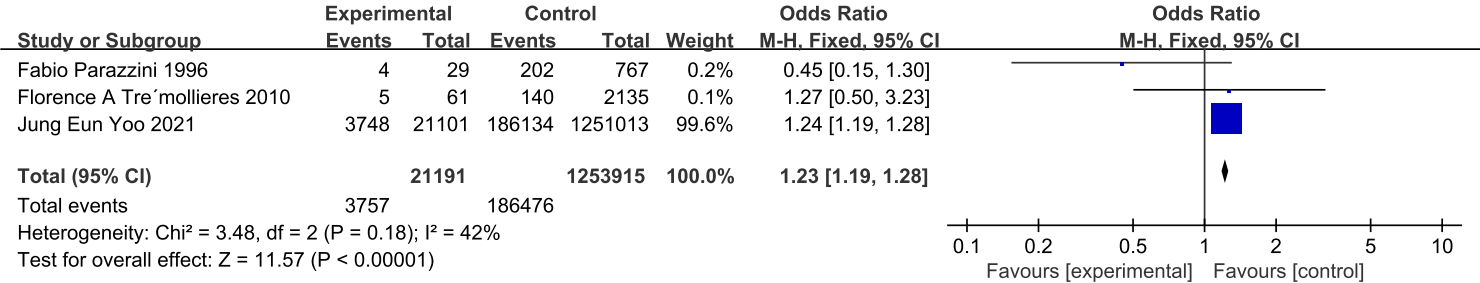

Supplement: Supplementary file 1 — Additional file 1. Forest plots of the main subgroups in the study. [file 13018_2023_4051_MOESM1_ESM.zip › Supplementary material/Age at menopause<40—Forest plot.pdf]

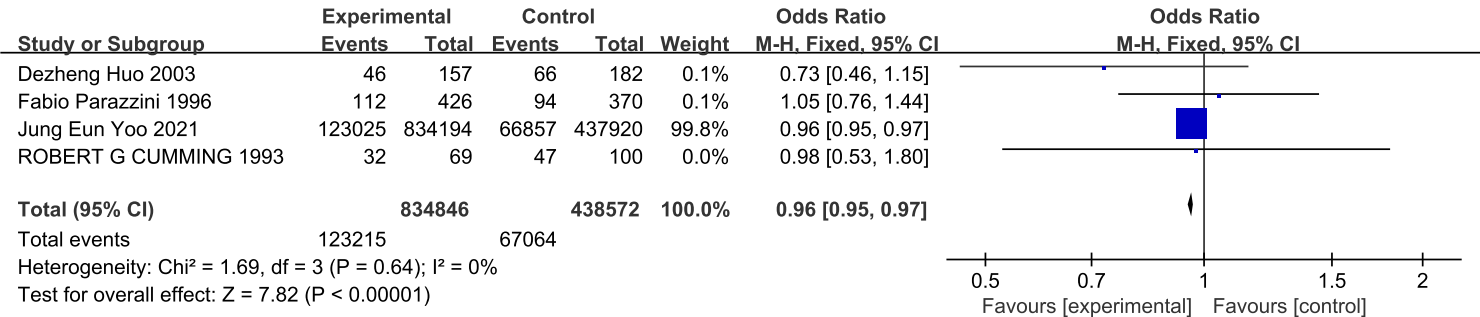

Supplement: Supplementary file 1 — Additional file 1. Forest plots of the main subgroups in the study. [file 13018_2023_4051_MOESM1_ESM.zip › Supplementary material/Age at menopause>50—Forest plot.pdf]

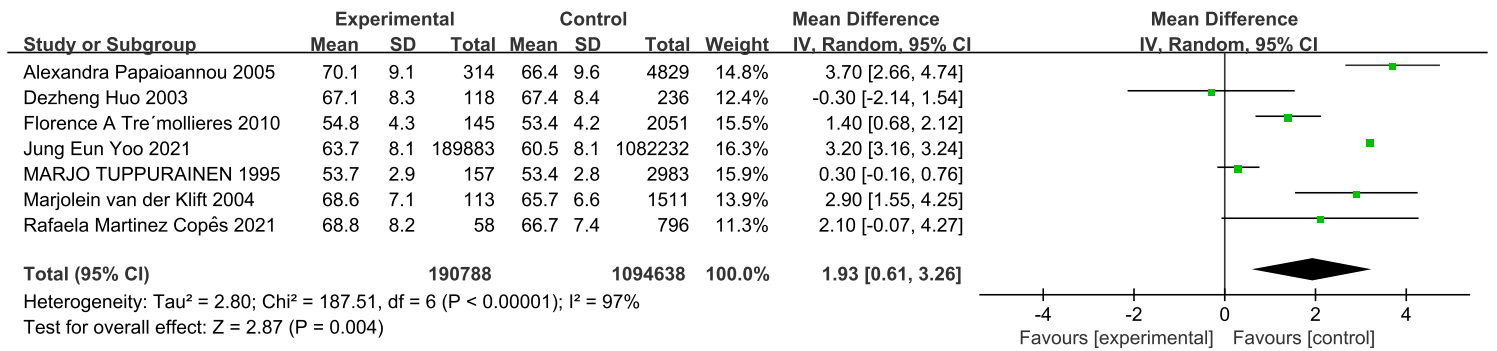

Supplement: Supplementary file 1 — Additional file 1. Forest plots of the main subgroups in the study. [file 13018_2023_4051_MOESM1_ESM.zip › Supplementary material/Age-Forest plot.pdf]

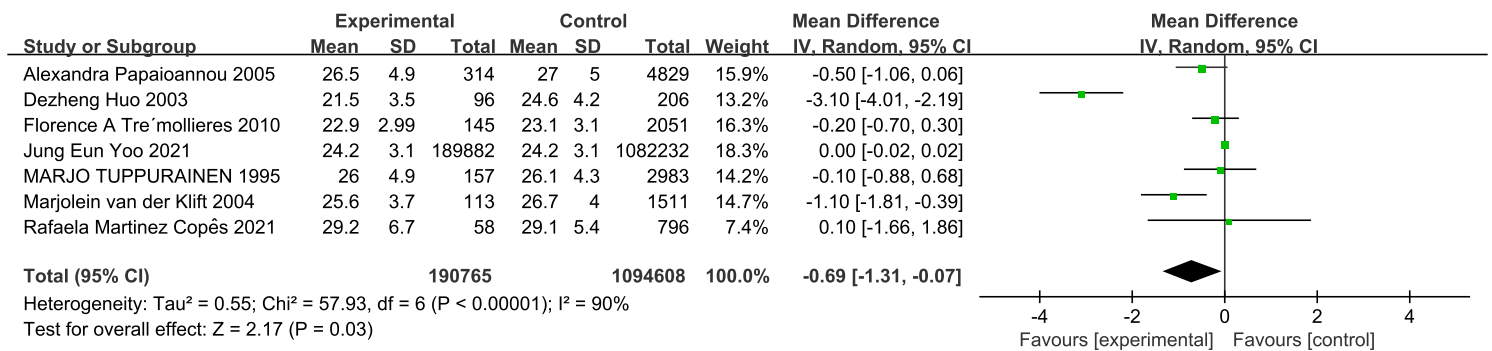

Supplement: Supplementary file 1 — Additional file 1. Forest plots of the main subgroups in the study. [file 13018_2023_4051_MOESM1_ESM.zip › Supplementary material/BMI—Forest plot.pdf]

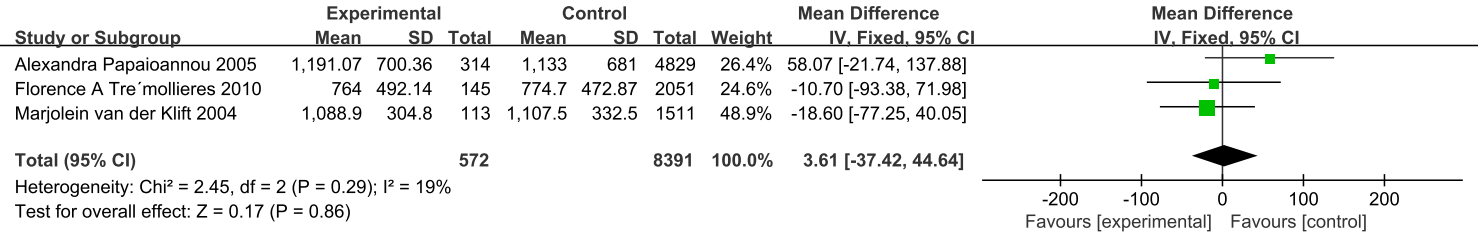

Supplement: Supplementary file 1 — Additional file 1. Forest plots of the main subgroups in the study. [file 13018_2023_4051_MOESM1_ESM.zip › Supplementary material/Calcium daily intake—Forest plot.pdf]

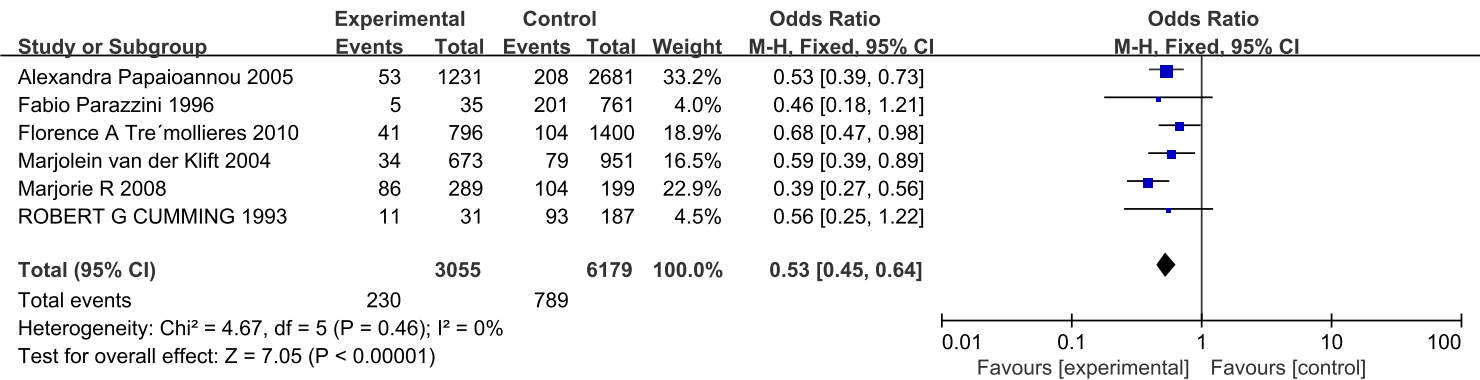

Supplement: Supplementary file 1 — Additional file 1. Forest plots of the main subgroups in the study. [file 13018_2023_4051_MOESM1_ESM.zip › Supplementary material/Estrogen use—Forest plot.pdf]

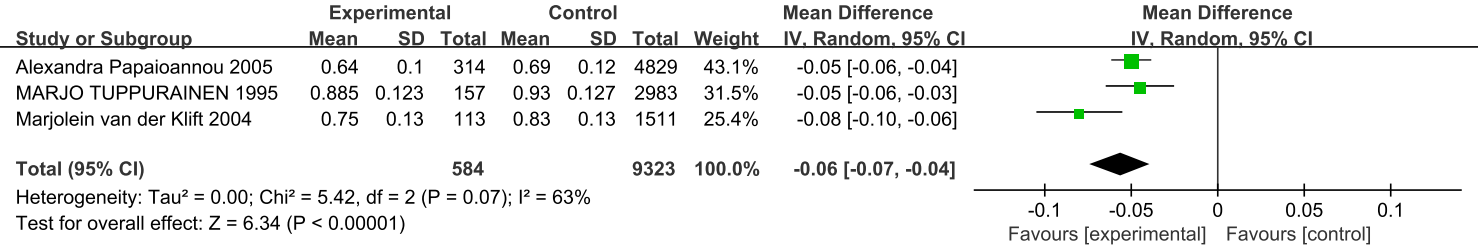

Supplement: Supplementary file 1 — Additional file 1. Forest plots of the main subgroups in the study. [file 13018_2023_4051_MOESM1_ESM.zip › Supplementary material/Femoral neck—Forest plot.pdf]

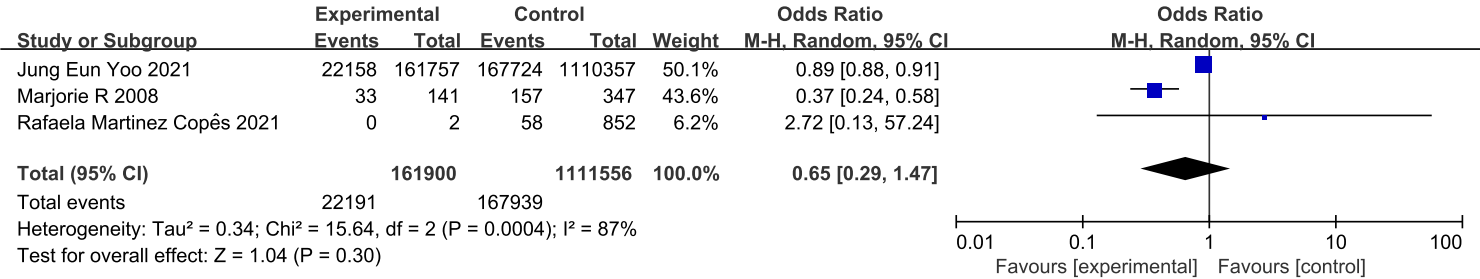

Supplement: Supplementary file 1 — Additional file 1. Forest plots of the main subgroups in the study. [file 13018_2023_4051_MOESM1_ESM.zip › Supplementary material/History of alcohol intake—Forest plot.pdf]

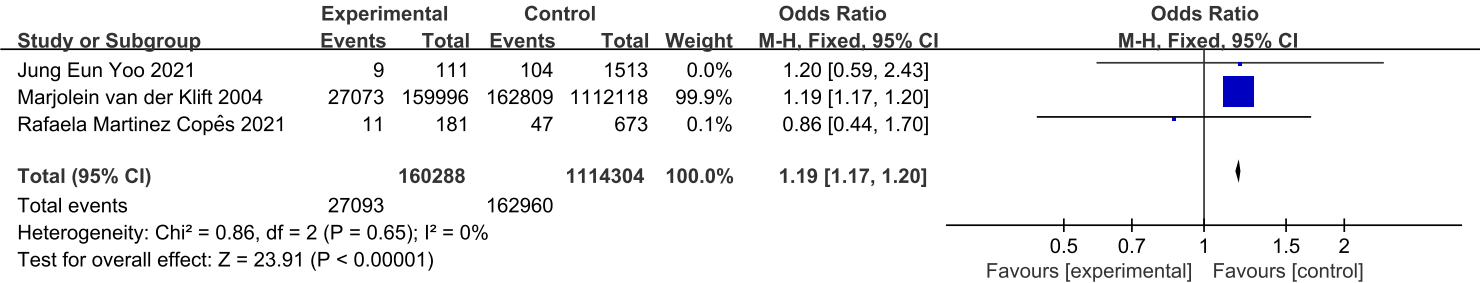

Supplement: Supplementary file 1 — Additional file 1. Forest plots of the main subgroups in the study. [file 13018_2023_4051_MOESM1_ESM.zip › Supplementary material/History of diabetes mellitus—Forest plot.pdf]

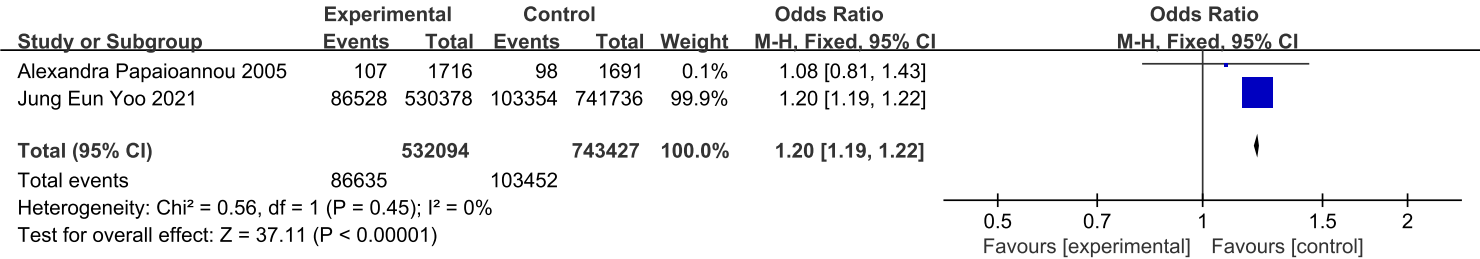

Supplement: Supplementary file 1 — Additional file 1. Forest plots of the main subgroups in the study. [file 13018_2023_4051_MOESM1_ESM.zip › Supplementary material/History of hypertension—Forest plot.pdf]

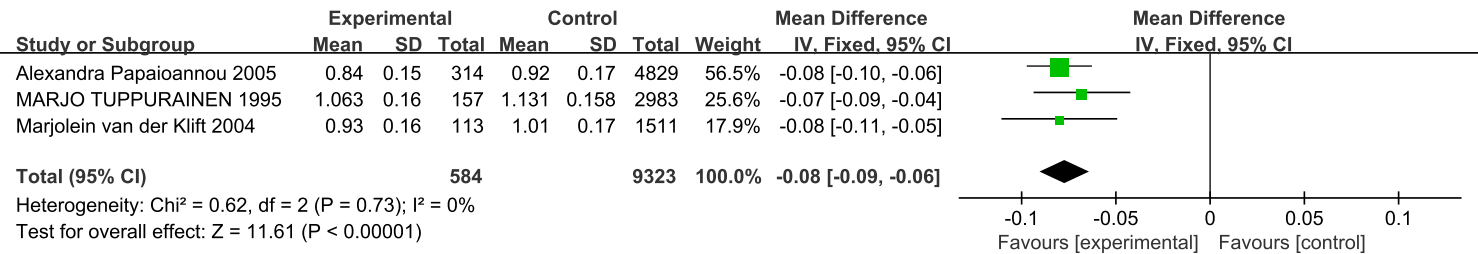

Supplement: Supplementary file 1 — Additional file 1. Forest plots of the main subgroups in the study. [file 13018_2023_4051_MOESM1_ESM.zip › Supplementary material/Lumbar spine—Forest plot.pdf]

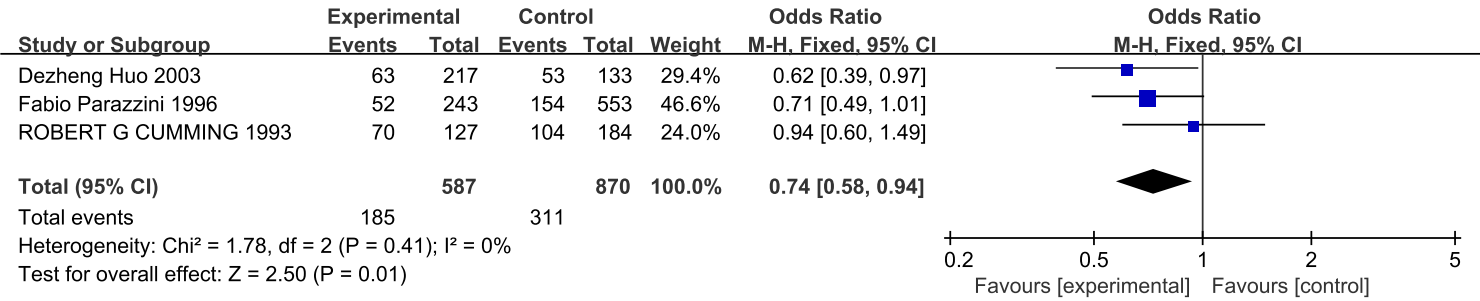

Supplement: Supplementary file 1 — Additional file 1. Forest plots of the main subgroups in the study. [file 13018_2023_4051_MOESM1_ESM.zip › Supplementary material/Parity≥3—Forest plot.pdf]

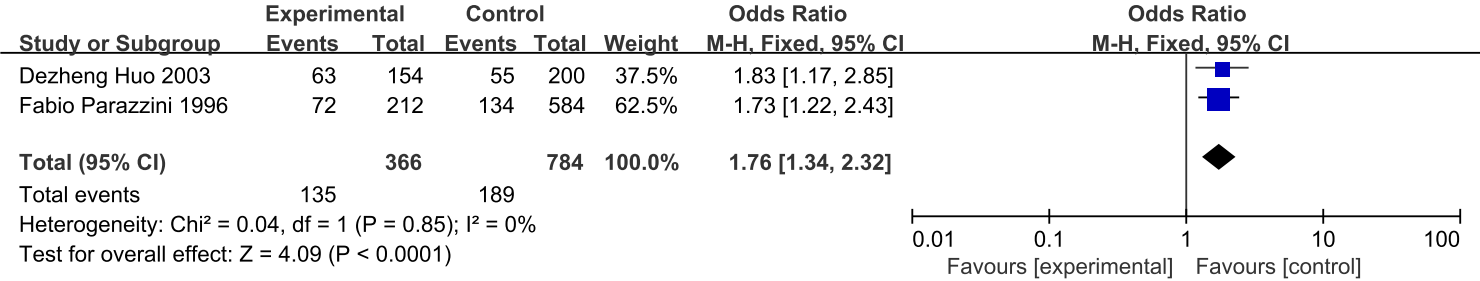

Supplement: Supplementary file 1 — Additional file 1. Forest plots of the main subgroups in the study. [file 13018_2023_4051_MOESM1_ESM.zip › Supplementary material/Senior high and above—Forest plot.pdf]

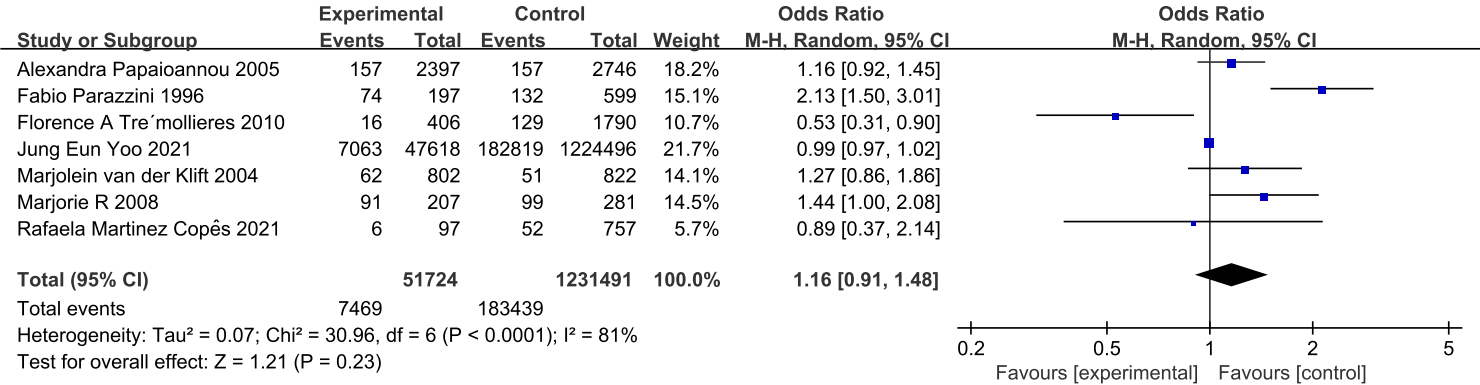

Supplement: Supplementary file 1 — Additional file 1. Forest plots of the main subgroups in the study. [file 13018_2023_4051_MOESM1_ESM.zip › Supplementary material/Smoking—Forest plot.pdf]

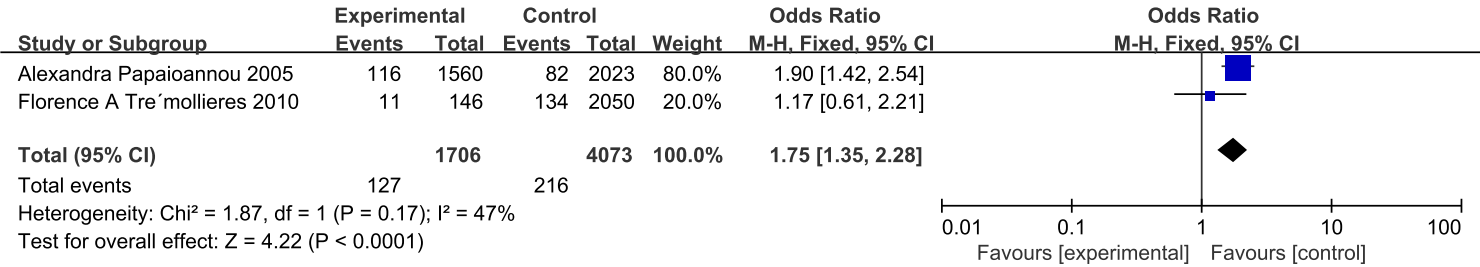

Supplement: Supplementary file 1 — Additional file 1. Forest plots of the main subgroups in the study. [file 13018_2023_4051_MOESM1_ESM.zip › Supplementary material/Vitamin D supplements—Forest plot.pdf]
